# Assessment of the pharmacokinetics and dynamics of two combination regimens of fosmidomycin-clindamycin in patients with acute uncomplicated falciparum malaria

**DOI:** 10.1186/1475-2875-7-225

**Published:** 2008-10-31

**Authors:** Ronnatrai Ruangweerayut, Sornchai Looareesuwan, David Hutchinson, Anurak Chauemung, Vick Banmairuroi, Kesara Na-Bangchang

**Affiliations:** 1 Mae Sot Hospital, Tak Province, Thailand; 2 Hospital for Tropical Diseases, Faculty of Tropical Diseases, Mahidol University, Bangkok, Thailand; 3 Jomaa Pharma GmbH, Schnackenburgallee 116A, 22525 Hamburg, Germany; 4 Faculty of Allied Health Sciences, Thammasat University, Pathumthani, Thailand

## Abstract

**Background:**

This study investigated the pharmacokinetics of fosmidomycin when given in combination with clindamycin at two dosage regimens in patients with acute uncomplicated falciparum malaria.

**Methods:**

A total of 70 patients with acute uncomplicated *Plasmodium falciparum *malaria who fulfilled the enrolment criteria were recruited in the pharmacokinetic study. Patients were treated with two different dosage regimens of fosmidomycin in combination with clindamycin as follows:

Group I: fosmidomycin (900 mg) and clindamycin (300 mg) every 6 hours for 3 days (n = 25); and Group II: fosmidomycin (1,800 mg) and clindamycin (600 mg) every 12 hours for 3 days (n = 54).

**Results:**

Both regimens were well tolerated with no serious adverse events. The 28-day cure rates for Group I and Group II were 91.3 and 89.7%, respectively. Steady-state plasma concentrations of fosmidomycin and clindamycin were attained at about 24 hr after the first dose. The pharmacokinetics of both fosmidomycin and clindamycin analysed by model-independent and model-dependent approaches were generally in broad agreement. There were marked differences in the pharmacokinetic profiles of fosmidomycin and clindamycin when given as two different combination regimens. In general, most of the dose-dependent pharmacokinetic parameters (model-independent C_max_: 3.74 *vs *2.41 μg/ml; C_max-ss_: 2.80 *vs *2.08 μg/ml; C_max-min-ss_: 2.03 *vs *0.71 μg/ml; AUC: 23.31 *vs *10.63 μg.hr/ml (median values) were significantly higher in patients who received the high dose regimen (Group II). However, C_min-ss _was lower in this group (0.80 *vs *1.37 μg/ml), resulting in significantly higher fluctuations in the plasma concentrations of both fosmidomycin and clindamycin following multiple dosing (110.0 vs 41.9%). Other pharmacokinetic parameters, notably total clearance (CL/F), apparent volume of distribution (V/F, V_z_/F) and elimination half-life (t_1/2z_, t_1/2e_) were also significantly different between the two dosage regimens. In addition, the dose-dependent pharmacokinetics of both fosmidomycin and clindamycin tended to be lower in patients with recrudescence responses in both groups.

**Conclusion:**

The findings may suggest that dosing frequency and duration have a significant impact on outcome. The combination of fosmidomycin (900 mg) and clindamycin (300–600 mg) administered every six hours for a minimum of five days would constitute the lowest dose regimen with the shortest duration of treatment and which could result in a cure rate greater than 95%.

## Background

The increasing devastation of malarial infections is currently responsible for hundreds of millions of clinical cases and several million deaths annually [1]. The *Plasmodium falciparum *parasite causing the disease is now becoming highly resistant to a wide variety of anti-malarial drugs including chloroquine and the sulphadoxine/pyrimethamine combination [1]. This underscores the urgent requirement for new effective and safe anti-malarial drugs with novel modes of action for the treatment of multidrug resistant *P. falciparum *malaria.

Fosmidomycin has been shown to be an effective blood schizonticide in addition to its wide spectrum of antibacterial activity. It possesses a novel mode of action through inhibition of 1-deoxy-*D*-xylulose *5*-phosphate (DOXP) reductoisomerase, an essential enzyme of the non-mevalonate pathway. This occurs in the organelles of the parasites, the apicoplasts, where it selectively blocks the biosynthesis of isopentenyl diphosphate and the subsequent development of isoprenoids in *P. falciparum *[2-5]. The drug possesses potent anti-malarial activity both *in vitro *and *in vivo*. However, in the treatment of clinical malaria, the high rate of recrudescent infections precludes its use as monotherapy [6]. The combination of fosmidomycin with clindamycin emerged as a potential anti-malarial treatment on the basis of *in vitro *and *in vivo *synergistic activity [7,8] and the outcome of subsequent clinical studies in Gabonese children [9,10].

The first clinical pharmacokinetic data from studies of fosmidomycin alone and in combination with clindamycin have recently been documented for 33 Thai patients diagnosed with acute uncomplicated *P. falciparum *malaria [11]. The study constituted part of two dose optimization Phase II trials of fosmidomycin monotherapy (1,200 mg every 8 hours for 7 days] and of combination therapy with clindamycin (fosmidomycin: 900 mg every 12 hours for 7 days; clindamycin: 600 mg every 12 hours for 7 days] [12,13]. Both the monotherapy and combination therapy regimens were well tolerated and no serious adverse events were reported. Combination therapy with fosmidomycin and clindamycin proved highly effective with a 100% cure rate, whereas the cure rate with the monotherapy regimen was only 22% during the 28-day follow up period.

The pharmacokinetics of fosmidomycin when administered alone and in combination with clindamycin were similar except for the apparent volumes of distribution (V_z_/F) and total clearances (CL/F) that were significantly lower for the combination regimen. Steady-state plasma concentrations of fosmidomycin and clindamycin were attained at the time of the second or third dose. There was no evidence of dose accumulation during multiple dosing. Urinary recovery of fosmidomycin was 18.7 and 20% following monotherapy and combination therapy respectively.

The present pharmacokinetic study aimed to optimize the regimen of fosmidomycin and clindamycin when administered over three days as an effective and safe treatment for multidrug-resistant *P. falciparum *malaria.

## Patients and methods

### Study design and patients

This pharmacokinetic study was part of an open-label, randomized uncontrolled, dose optimization clinical trial in 114 patients with acute uncomplicated falciparum malaria. The study was conducted during 2006 at the two main study sites – Bangkok (Hospital for Tropical Diseases; n = 84), and Tak Province (Mae Sot General Hospital and Umphang Hospital; n = 30). The latter is located in an area along the Thai-Myanmar border, where multidrug resistance exists. Approval of the study protocol was obtained from the Ethics Committees of the Ministry of Public Health of Thailand and the Faculty of Tropical Medicine, Mahidol University, Bangkok. A total of 80 patients, 77 males and 3 females, of varying ethic groups (30 Karen, 20 Burmese and 20 Thai) were included in the pharmacokinetic study.

The age, body weight and admission parasitaemia ranges were 15 – 61 years, 45.0 – 68.0 kg and 1,152 – 254,800 asexual parasites/μl respectively. Pre-treatment investigations consisted of clinical assessments (general medical history, demographic data, significant medical history, previous drug administration, physical examinations, monitoring of vital signs and malaria signs and symptoms), laboratory assessments (thick and thin blood smears for parasite identification/quantification), routine haematology and biochemistry, urinalysis and stool examination for parasites and ova). Most patients showed baseline laboratory parameters outside the normal ranges, which are common in patients with acute falciparum malaria and all were mild or moderate in severity.

All patients were admitted to the hospitals for 7 days and were requested to attend for follow-up visits on days 14, 21 and 28 or until suspected signs and/or symptoms of malaria appeared.

### Drug administration

This study was planned as a proof-of-concept study to investigate the combination regimen of fosmidomycin-clindamycin that would produce approaching 100% cure rate with short duration of 3 days and less frequent dosing interval. The design of dosage regimens used in the present study was based on background information from previous studies [11-13]. The total number of patients recruited for Group I and II was at the ratio of approximately 1: 3.5 (25 *vs *89 cases, respectively). Patients from each study site were sequentially entered into the study and treated with one of two different combination regimens of fosmidomycin and clindamycin as follows: Group I (n = 25): fosmidomycin sodium capsules (Alphamed PHARBIL Arzneimittel GmbH, Germany) 450 mg × 2 tablets, six-hourly for 3 days plus clindamycin hydrochloride capsules (Alphamed PHARBIL Arzneimittel GmbH, Germany) 150 mg × 2 tablets, six-hourly for 3 days; and Group II (n = 89): fosmidomycin sodium capsules 450 mg × 4 tablets, twelve-hourly for 3 days plus clindamycin hydrochloride capsules 150 mg × 4 tablets, twelve-hourly for 3 days.

The patients fasted overnight (8–10 hr) and emptied their bladders immediately before drug administration. The drugs were administered orally with 250 ml of water and a standard hospital meal (20–25% fat content) under supervision of the assigned study staff. After ingestion of the medication, patients were observed for 1 hr and in the event of vomiting, the medication was re-administered. Meals were provided at 4 hr post-dosing. Fruit juice and water were freely available throughout the period of hospitalization. The administration of prescription drugs, deemed necessary for the well-being of the patients, were permitted during the study period and documented.

### Blood and urine collection

Pharmacokinetic investigation was performed in all (n = 25) patients in Group I, while only 54 cases in Group II from both study sites were randomly recruited for pharmacokinetic investigation. Blood samples (4 ml) were obtained at the following time points:- Group I: 0, 1, 2, 3, 4, 6, 8, 12, 18, 24, 26, 30, 36, 42, 48, 50, 54, 60, 66, 72 and 74 hours from the commencement of dosing; Group II: 0, 1, 2, 3, 4, 6,12, 14,24, 26, 36, 38, 48, 50, 60, 62, 72 and 74 hours from the commencement of dosing. Blood samples were drawn via an indwelling venous cannula and collected in heparinized tubes. Where appropriate, blood samples were taken immediately prior to the next dose. Immediately after collection, the tubes were centrifuged (2,000 g) for 10 minutes. The plasma samples were stored at -80°C pending analysis.

Twelve hourly urine collections were made during the first four days as follows: 0–12, 13–24, 25–36, 37–48, 49–60, 61–72, 73–84 and 85–96 hours. The total volume of each collection was recorded. A 20 ml aliquot was taken from each collection and stored at -80°C until analysis.

### Efficacy assessment

Efficacy assessments included clinical and parasitological evaluations. Clinical signs/symptoms of malaria including body temperature were monitored on days 0, 1, 2, 3, 4, 5, 6, 7, 14, 21 and 28. Finger-prick blood smears were examined at 6 hourly intervals until three consecutive negative readings were obtained, then once daily during hospitalization, and then on days 14, 21 and 28. The films were stained with Giemsa and parasite counts were determined by counting the number of asexual parasites per 1,000 red blood cells on a thin film or per 200 white blood cells on a thick film. The protocol used to determine the response to treatment was adapted from the World Health Organization [14]. Efficacy was assessed using the following criteria: (i) polymerase chain reaction (PCR)-adjusted 28 day cure rate:- the proportion of patients with clearance of asexual parasitaemia within 7 days of the commencement of treatment and without subsequent recrudescence during the 28 day follow-up period: (ii) parasite clearance time (PCT):- as calculated from the commencement of therapy to the time of the first negative film for asexual parasites subject to two consecutive films remaining negative: (iii) fever clearance time (FCT):- as calculated from the commencement of therapy to the time that the temperature falls below 37.5°C and remains below 37.5°C for 48 hours.

### Safety and tolerability assessment

Safety and tolerability were assessed on the clinical findings and abnormal laboratory tests (routine haematology and biochemistry) that first occur or increase in severity during the study period in accordance with the Common Toxicity Criteria CTC grade [15].

### Drug analysis

Fosmidomycin concentrations in plasma and urine were determined by a bioassay system based on agar disk diffusion using *Enterobacter cloacae *ATCC 23355 as the test organism [16]. The precision of the assay method was examined and the within-day (repeatability) and day-to-day (reproducibility) variations were below 5% (% coefficient of variation: % C.V.). Good accuracy was also observed for both the intra-day and inter-day assays. The limit of quantification was 1 ng using 40 μl of plasma or 7.5 μl of urine.

Plasma concentrations of clindamycin were measured by high performance liquid chromatography as described previously [17]. The precision of the method within-day and day-to-day variations were below 15% (% C.V.) Good accuracy was observed for both the intra-day and inter-day assays, as indicated by the minimal deviation of mean values found with measured samples compared to theoretical values (below ± 15%). The limitation of quantification was considered acceptable at 0.07 μg, using plasma sample quantities of 1 ml.

### Pharmacokinetic analysis

The non-compartmental pharmacokinetic analysis was used to determine the pharmacokinetic parameters of fosmidomycin and clindamycin after a single initial dose of the two drugs [18]. *C*_max _was defined as the maximum plasma concentrations and *t*_max _was the time required to reach maximum plasma concentrations following a single initial dose or the final dose. C_min-obs _was the observed trough concentration. The area under the concentration-time curve from the zero time point to the last sampling point yielding a quantifiable concentration (AUC_0, t_) was calculated with the log-linear trapezoidal method. A minimum of three final sampling points were used to determine the first order constants generated by the terminal portion of the curve (λ_z_). Terminal phase elimination half-life (t_1/2z_) was defined as t_1/2z _= ln(2)/λ_z_. The AUC from time zero to infinity (AUC) was calculated as AUC_0-t _+ Ct/λ_z_, where C_t _stands for the last measurable concentration. Total clearances (CL/F) were calculated from Dose/AUC_0,8 _and apparent volumes of distribution (V_z_/F) were calculated as CL/F/λ_z_, where F is bioavailability. The maximum plasma concentrations at steady-state (C_max-ss_), minimum plasma concentrations at steady-state (C_min-ss_) and average plasma concentrations at steady-state (C_ave-ss_) following a fast release oral formulation could all estimated using:

$$C_{\max-\text{ss}}=\frac{\text{Dose}}{V_{\text{z}}F(1-{\text{e}}^{-\lambda \cdot \tau })}$$

C_min-ss _= C_max-ss _·e^-λ.τ^

$${\text{C}}_{\text{ave-ss}}=\frac{\text{Dose}}{\text{CL}/\text{F}\cdot \tau }$$

Where *τ *is the dosing interval (hr).

Fluctuations between C_max-ss _and C_min-ss _were calculated from the ratio between the two parameters.

For the purpose of future stimulation and prediction as well as pharmacokinetic/pharmacodynamic modelling, the compartment open model (one- or two-) with first-order absorption and elimination with absorption lag time was fitted to the data by iterative, weighted non-linear regression using ADAPT II (release 4.0) [19]. The observed concentrations of fosmidomycin and clindamycin were weighted as the reciprocal of the analytical variance. Data reported in the previous study [11] were used for the initial estimation of the pharmacokinetic parameters. The adequacy of the pharmacokinetic models chosen was based on statistical methods for assessing the validity of the models for describing the concentration-time data, i.e., F-ratio test, Akaike information criterion (AIC), Schwartz and Imbimbo criteria.

Total urinary excretion of fosmidomycin was calculated from the sum of amounts of drug excreted during each 12-hour interval multiplied by total urine volume at each interval. The dose excreted (%) was calculated from total amounts of drug excreted in urine during 72 hours divided by total dose given.

### Statistical analysis

Descriptive statistics were calculated for the pharmacokinetic variables. Geometric means were determined for baseline parasite counts. All pharmacokinetic parameters were presented as median values (95% C.I.). Comparisons of the pharmacokinetic parameters between the two groups were performed using the Mann-Whitney U test. The level of statistical significance was set at α = 0.05 for all tests.

## Results

**Figure 1:**
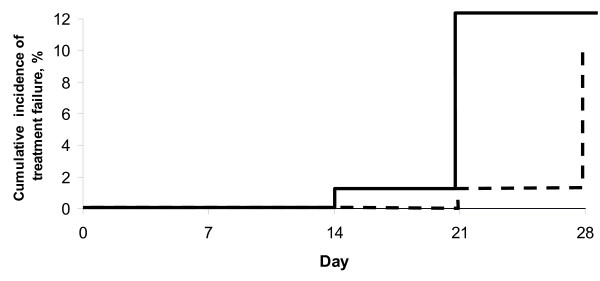
Kaplan-Meier curve of PCR-adjusted cumulative treatment failure over the 28-day follow-up period.

A total of 25 (Group I) and 89 (Group II) patients received treatment with the two different combination regimens of fosmidomycin and clindamycin. Most patients had baseline laboratory parameters (haematology and serum biochemistry) outside the normal ranges; most are common in patients with acute falciparum malaria and all were mild or moderate in severity. For Group I, one case was withdrawn after eight doses due to hyperpyrexia; one case was excluded for efficacy analysis due to appearance of *P. vivax *in peripheral blood smear. Two cases had reappearance of *P. falciparum *in their blood smears on day 28 and both were confirmed by PCR as recrudesced parasites. For Group II, three cases were withdrawn (one case after two doses due to allergy, one case each after four and five doses due to diarrhoea; eight cases were excluded for efficacy analysis due to appearance of *P. vivax *in peripheral blood smear (two cases on day 14, one case on day 20 and five cases on day 21). Seven cases had reappearance of *P. falciparum *in their blood smears during days 21–28 and all but one was confirmed by PCR as recrudesced parasites. PCR-adjusted cure rates on day 28 for Group I and II were 91.3% (21/23) and 89.7% (70/78) respectively. Kaplan-Meier curve of PCR-adjusted cumulative treatment failure over the 28-day follow-up period is shown in Figure 1.

FCTs and PCTs [median values] were 40 (4–76) and 50 (20–80) hours respectively. Concomitant medication included paracetamol, dimenhydinate, diphenhydramine and chlorpheniramime. Both combination regimens were well tolerated with no serious adverse events. Gastro-intestinal symptoms were the most commonly reported adverse experiences with abdominal pain developing in 10% of patients, diarrhoea in 5% and vomiting in 4%.

### Pharmacokinetics and urinary excretion

**Figure 2:**
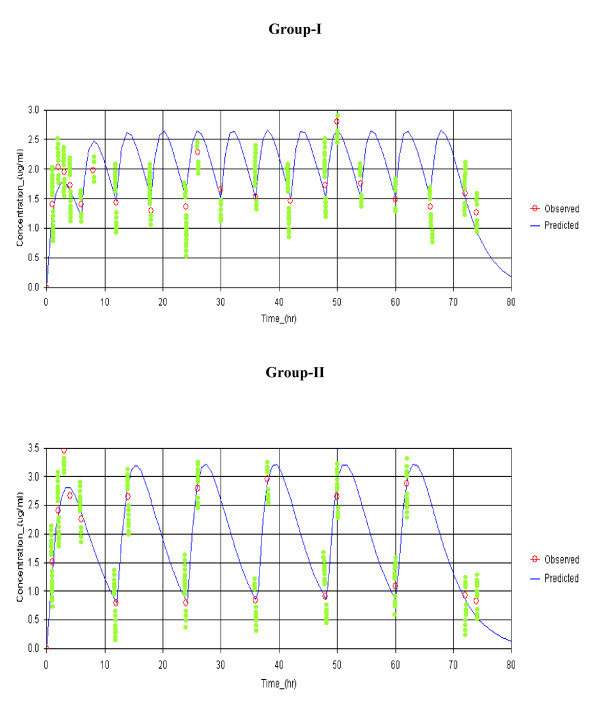
**Observed and predicted plasma concentration-time profiles (median values) of fosmidomycin in patients with sensitive responses in Group-I (fosmidomycin 900 mg plus 300 clindamycin every 6 hours for 3 days; N = 23) and Group-II (fosmidomycin 1800 mg plus 600 mg clindamycin every 12 hours; N = 45).** The curve represents simulated concentration-time profile based on the median concentrations in each group.

**Figure 3:**
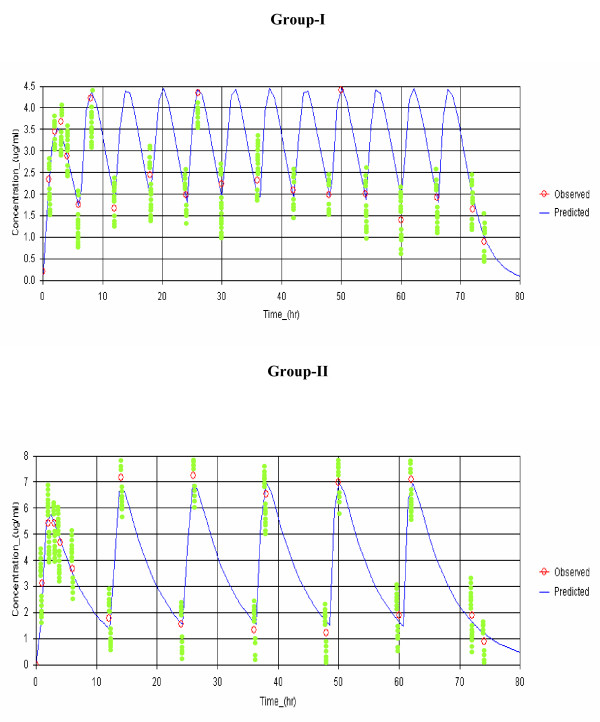
**Observed and predicted plasma concentration-time profiles (median values) of clindamycin in patients with sensitive response in Group-I (fosmidomycin 900 mg plus 300 clindamycin every 6 hours for 3 days) and Group-II (fosmidomycin 1800 mg plus 600 mg clindamycin every 12 hours).** The curve represents simulated concentration-time profile based on the median concentrations in each group.

The concentration-time data of both fosmidomycin and clindamycin were in all cases best fitted with one-compartment open model with first-order absorption and elimination. Observed and model-predicted (one-compartment model with first-order absorption and elimination with absorption lag time) median plasma concentrations of fosmidomycin and clindamycin in patients with sensitive responses following implementation of the two combination regimens are shown in Figures 2 and 3, respectively. The pharmacokinetic parameters of both drugs analysed by model-independent and model-dependent methods are summarized in Table 1 and 2 respectively. Variability as expressed as % C.V. for all parameters varied between 7.7 and 40.5% for fosmidomycin and 12.2 and 38.9% for clindamycin. Results obtained from model-dependent analysis for both fosmidomycin and clindamycin were generally in agreement with those obtained from model-independent analysis. Fluctuation was significantly higher (260% for fosmidomycin and 120% for clindamycin) following the administration of the high dose combination regimen.

**Table 1:** Pharmacokinetics of fosmidomycin analysed by model-independent and model-dependent approach in patients with sensitive responses in Group-I and II.

Pharmacokinetic Parameters	**Model-independent**		**Model-dependent**	
	**Group-I** **(n = 23)**	**Group-II** **(n = 45)**	**Group-I** **(n = 23)**	**Group-II** **(n = 45)**
t_max _(hr)	2.0 (1.0–4.0)	3.0 (1.0–6.0)	2.6 (1.0–3.8)^a^	3.2 (0.8–4.3)
t_lag _(hr)	NA	NA	0.3 (0.001–0.8)	0.55 (0.001–1.6)
K_01 _(/hr)	NA	NA	0.35 (0.15–3.00)	0.56 (0.09–4.00)
t_1/2a _(hr)	NA	NA	2.0 (0.2–8.0)	1.97 (0.1–7.7)
C_max _(μg/ml)	2.41 (0.91–16.23)^b^	3.74(1.49–8.94)	1.77 (0.96–2.97)^c^	2.92 (1.40–6.93)
AUC (μg.hr/ml)	10.63 (4.27–53.85)	23.31 (11.42–66.13)	13.30 (6.89–24.33)^d^	26.18 (14.72–54.49)
K_10 _(/hr)	NA	NA	0.37 (0.09–1.92)	0.36 (0.08–2.00)
t_1/2z _(hr)	3.5 (0.8–14.9)^e^	4.2 (2.1–7.9)	NA	NA
t_1/2e _(hr)	NA	NA	1.6 (0.2–5.3)^f^	2.2 (0.3–8.2)
CL/F (l/hr)	47.51 (16.46)^g^	57.69 (9.29–132.54)	67.62 (23.39–130.57)	68.27 (33.03–122.31)
Vz/F (l)	272.64 (20.22–702.25)^h^	338.56 (131.65–895.17)	NA	NA
V/F (l)	NA	NA	194.50 (34.17–855.40)	174.11 (30.23–924.62)
C_min-ss _(μg/ml)	1.37 (1.08–5.09)^i^	0.80 (0.20–2.11)	1.62 (0.66–2.69)^j^	0.97 (0.20–2.25)
C_max_-_ss _(μg/ml)	2.28 (1.34–9.43)^k^	2.80 (1.18–6.52)	2.63 (1.46–4.64)^l^	3.26 (1.83–7.27)
C_ave-ss _(μg/ml)	1.77 (0.71–8.98)	1.94 (0.95–5.51)	2.22(1.45–4.06)	2.18(0.51–4.54)
C_max-min-ss _(μg/ml)	0.71 (0.05–4.34)^m^	2.03 (0.59–5.41)	1.11 (0.39–2.23)^n^	2.24 (0.04–5.76)
Fluctuation (%)	41.9 (40.1–203.6)°	110.0 (30.8–223.4)	49.2 (50.8–191.5)^p^	118.7 (41.2–218.5)

Data are presented as median (95% CI).

NA = Not applicable

^a ^Statistically significant difference from Group-II with p = 0.009, 95%CI = 0.9 (0.2–1.5); ^b ^p = 0.029, 95%CI = 1.45(0.77–2.04); ^c ^p < 0.0001, 95%CI = 0.78(0.15–2.11); ^d ^p < 0.0001, 95%CI = 15.99(4.15–26.77); ^e ^p = 0.022, 95%CI = 0.8(0.4–6.5),;^f ^p < 0.0001, 95%CI = 0.2(0.09–0.9); ^g ^p = 0.006, 95%CI = 14.77(6.11–122.14); ^h ^p = 0.0001, 95%CI = 76.66(33.48–48.77); ^i ^p = 0.0001, 95%CI = 0.78(0.34–2.25); ^j ^p < 0.0001, 95%CI = 0.32(0.11–1.02); ^k ^p = 0.048, 95%CI = 0.45(0.20–0.79); ^l ^p ≤ 0.0001, 95%CI = 0.98(0.45–1.45); ^m ^p = 0.033, 95%CI = 0.64(0.23–1.78); ^n ^p = 0.039, 95%CI = 0.67(0.23–1.12); ° p = 0.001, 95%CI-65.8(23.1–102.3); ^p ^p = 0.0001, 95%CI = 62.1(24.6–123.6) (Mann-Whitney U-test)

**Table 2:** Pharmacokinetics of clindamycin analysed by model-independent and model-dependent approach in patients with sensitive responses in Group-I and II.

Pharmacokinetic Parameters	**Model-independent**		**Model-dependent**	
	**Group-I** **(n = 23)**	**Group-II** **(n = 45)**	**Group-I** **(n = 23)**	**Group-II** **(n = 45)**
t_max _(hr)	2.0 (1.0–4.0)	3.0 (1.0–4.0)	2.0 (0.7–3.0)	2.4 (0.6–4.4)
t_lag _(hr)	NA	NA	0.44 (0.00–0.90)	0.67 (0.001–1.45)
K_01 _(/hr)	NA	NA	0.62 (0.40–4.00)	1.48 (0.11–4.00)
t_1/2a _(hr)	NA	NA	1.1 (0.1–1.7)	0.4 (0.1–6.0)
C_max _(μg/ml)	4.34 (0.89–8.23)^a^	6.23 (3.23–25.11)	3.70 (1.15–5.61)^b^	5.79 (3.38–18.11)
AUC (μg.hr/ml)	16.82 (4.07–31.46)^c^	39.77 (3.41–136.16)	21.13 (8.91–37.40)^d^	52.91 (28.81–126.73)
K_10 _(/hr)	NA	NA	0.40 (0.15–0.78)	0.18 (0.09–0.68)
t_1/2z _(hr)	2.7 (0.7–7.7)^e^	4.4 (1.8–11.5)	NA	NA
t_1/2e _(hr)	NA	NA	1.0 (0.8–4.7)^f^	3.9 (1.0–7.7)
CL/F (l/hr)	11.81 (4.70–68.56)	11.00 (2.11–332.66)	14.19 (8.02–33.68)	11.85 (4.73–42.31)
Vz/F (l)	51.99 (11.45–207.07)^g^	72.82 (24.94–896.94)	NA	NA
V/F (l)	NA	NA	38.12 (26.37–229.06)^h^	78.85 (17.85–178.04)
C_min-ss _(μg/ml)	1.99 (0.87–4.56)	1.54 (0.23–6.98)	2.24 (0.97–24.00)	1.66 (0.19–4.93)
C_max_-_ss _(μg/ml)	4.34 (2.10–14.00)^i^	7.23 (3.45–16.54)	4.80 (1.00–8.47)^j^	6.80 (4.10–17.77)
C_ave-ss _(μg/ml)	2.80 (0.68–5.24)	3.31 (0.28–11.35)	1.88 (0.74–5.09)^k^	4.41 (2.40–10.56)
C_max-min-ss _(μg/ml)	2.39 (0.22–9.88)^l^	5.78 (1.89–13.33)	2.70 (0.86–4.13)^m^	4.96 (2.46–14.80)
Fluctuation (%)	104.4 (5.3–188.4)^n^	126.2 (29.2–145.6)	98.7 (12.9–201.2)°	158.4 (35.7–159.2)

Data are presented as median (95% CI).

NA = Not applicable

^a ^Statistically significant difference from Group-II with p < 0.0001, 95%CI = 1.56(0.80–3.70); ^b ^p < 0.0001, 95%CI = 2.01(0.88–4.10); ^c ^p = 0.0001, 95%CI = 15.46(8.11–19.23); ^d ^p < 0.0001, 95%CI = 15.45(9.11–20.23); ^e ^p = 0.0001, 95%CI = 1.5(0.4–1.3);^f ^p = 0.003, 95%CI = 0.8(0.4–4.3); ^g ^p = 0.001, 95%CI = 12.65(8.77–20.23); ^h ^p = 0.001, 95%CI = 20.14(15.22–50.47); ^i ^p < 0.0001, 95%CI = 2.23(0.89–4.55); ^j ^p < 0.0001, 95%CI = 1.56(0.89–4.55); ^k ^p < 0.0001, 95%CI = 1.12(0.44–2.36); ^l ^p = 0.007, 95%CI = 0.78(0.55–1.43); ^m ^p < 0.0001, 95%CI = 2.02(1.55–4.23); ^n ^p = 0.039, 95%CI = 10.7(2.0–25.1); ° p < 0.0001, 95%CI= 32.2(14.1–50.8) (Mann-Whitney U-test)

#### Fosmidomycin

All of the dose-dependent pharmacokinetic parameters including C_max_, C_max-ss_, C_max-min-ss _and AUC were significantly higher in Group II than for Group I (160, 120, 240 and 162% of that in Group I respectively). On the other hand, C_min-ss _was significantly lower in Group-II compared to Group I. Furthermore, total clearances (CL/F) and volumes of distribution (V/F, V_z_/F) were found to be significantly greater and t_1/2e _was significantly longer in Group II patients with all values being 120%. Total urinary excretion of fosmidomycin and the % dose excreted in Group II were about 68% of Group I: however a statistically significant difference was only observed with the % dose excreted.

#### Clindamycin

All dose-dependent pharmacokinetic parameters including C_max_, C_max-ss_, C_max-min-ss_, C_ave-ss _and AUC were significantly higher in Group II compared with Group I (150, 152, 210 and 240% of those in Group I respectively). In addition, V/F values were significantly larger (210%) and t_1/2e _longer (390%) in Group II patients. Moreover, V_z_/F and t_1/2e _were found to be significantly higher in Group II (210 and 390%, respectively).

### Relationship between plasma drug concentration and therapeutic outcome

#### Fosmidomycin

Based on the model-independent approach, all the dose-dependent parameters in Group II were lower in patients with recrudescent infections (n = 9) compared with those who were cured (n = 45), but statistically significant differences were observed only with C_max_, AUC, C_ave-ss _and C_max-min-ss _(50, 60, 80 and 60%, respectively) in patients who recrudesced compared to those who were cured. In addition, CL/F was significantly higher (210%) in recrudescent patients.

#### Clindamycin

One of the two patients who recrudesced in Group I had markedly lower plasma levels (AUC 24.94 μ.hr/ml, C_max _5.61 μ/ml, C_max-ss _5.06 μ/ml, C_min-ss _1.71 μ/ml), whereas another had markedly higher values for all of the dose-dependent pharmacokinetic parameters (AUC 4.52 μ.hr/ml, 2-hour concentration 0.68 μg/ml, C_max _1.12 μg/ml, C_max-ss _0.98 μg/ml, C_min-ss _0.54 μg/ml, C_ave-ss _0.77 μg/ml) when compared with the median values observed in the sensitive group. However, all of the dose-dependent parameters were lower in the Group II patients who recrudesced including C_ave-ss_, but statistically significant differences were only observed with C_max_, C_max-ss _and C_min-ss _(120, 150 and 140%, respectively) in patients with recrudescent infections compared with those who were cured. In addition, CL/F was significantly higher (210%). Fluctuation appeared to be higher (113%) in the group with recrudescence responses.

## Discussion

In the present study, the pharmacokinetic parameters of fosmidomycin and clindamycin which were obtained from model dependent and model-independent analysis were generally in broad agreement and with our recent investigation following the lower dose regimen (900 mg fosmidomycin plus 600 mg clindamycin given every 12 hours over 12 hours) [11]. Due to the marked variation in the model-fitted data, statistically significant differences between the two dosage regimens (Group I *vs *Group II and sensitive vs recrudescence responses) for some of the parameters was not observed. The dose-dependent pharmacokinetic parameters (elimination half-life, clearance, volume of distribution) for both fosmidomycin and clindamycin were similar with that reported in the previous study [11]. However, the dose-dependent pharmacokinetic parameters (C_max_, C_max-ss_, C_min-ss _and C_ave-ss_) of fosmidomycin observed in this study were markedly lower than that observed in the previous study even with twice as high dose of fosmidomycin (1,800 mg) was administered. C_ave _was only about 60 μg/ml in Group I patients in current study who received the same dose of 900 mg fosmidomycin. When comparing between Group I and Group II in the present study, almost all of the dose-dependent pharmacokinetic parameters of fosmidomycin and clindamycin (C_max_, C_max-ss_, C_min-ss_, C_ave-ss_, AUC) were significantly higher in the high dose Group II regimen showing varying degrees of elevation ranging from 120 to 280%. Since C_min-ss _was lower in the high dose group, fluctuation (%) was also greater in the high dose regimen. In addition, the dose-independent pharmacokinetic parameters, *i.e*., CL/F, V_z_/F and t_1/2z _or t_1/2e _were significantly greater in this group. The extent of the changes in these pharmacokinetic parameters was more pronounced with fosmidomycin than with clindamycin. These findings suggest that saturation in some of the pharmacokinetic processes for fosmidomycin occurred particularly with respect to elimination as renal clearance is the major route of drug elimination. This supposition is supported by the urinary excretion data which shows that the total urinary excretion of fosmidomycin was approximately 50% in Group II compared with Group I.

There was a relationship between plasma drug concentrations and therapeutic outcome since the dose-dependent pharmacokinetic parameters of fosmidomycin and clindamycin were significantly lower in the group of patients with recrudescence responses, particular in Group II patients. The extent of the differences in these pharmacokinetic parameters was more pronounced for fosmidomycin. Together, clinical efficacy and pharmacokinetics of fosmidomycin and clindamycin observed in the present study and in previous studies [11,13] suggest that the combination of fosmidomycin with fosmidomycin may provide an alternative treatment for multidrug resistant uncomplicated *Plasmodium falciparum *malaria in preference to the combination of artesunate/mefloquine. There has not been any prior evidence for cross resistance between fosmidomycin and other anti-malarials. Clindamycin is the most appropriate combination partner.*In vitro *studies in our laboratory confirm the pronounced antagonistic effect of fosmidomycin and the artemisinin derivatives [20]. Three dose regimens of this combination have been investigated for their efficacy and pharmacokinetics in studies Thailand. The first combination regimen of fosmidomycin (900 mg every 12 hr for seven days) plus clindamycin (600 mg every 12 hr for seven days) resulted in a 28-day cure rate of 100% [11,13]. In the present study, the combination of fosmidomycin (900 mg every 6 hr for 3 days *vs *1800 mg every 12 hr for 3 days) plus clindamycin (300 mg every 6 hr for 3 days *vs *600 mg every 12 hr for 3 days) resulted in a 28-day cure rates of 91.3 *vs *89.7%, respectively. Both combination regimens were well tolerated without any sign of serious adverse events. All of the observed adverse events were of mild or moderate in severity and are commonly associated with malaria infection. These findings may suggest that dosing frequency and duration have a significant impact on outcome. The combination of fosmidomycin (900 mg) and clindamycin (300–600 mg) given at 6 hourly for 7 days would be an ideal regimen, but is impractical for reasons of compliance. Based on the observed pharmacokinetic profiles, increasing the doses of fosmidomycin to 1800 mg and with a less frequent dosing interval of twelve hours resulted in marked fluctuations in the trough and peak plasma concentrations during steady-state. The importance of optimal fosmidomycin dose regimen in malaria treatment has been shown in the previous study [11]. Unlike fosmidomycin, the anti-malarial action of fosmidomycin is time-dependent [21,22]. Since the minimum inhibitory concentration (MIC) of fosmidomycin for multidrug resistant strains of *P. falciparum *is not known, it would be important to maintain the MIC as high as possible throughout at least three or four cycles of asexual replication. Nevertheless, for the partner drug clindamycin which its anti-malarial action is believed to be concentration-dependent [23], this would be a major gap in applying pharmacokinetics in designing of appropriate dose regimen in the combination regimen. Parasite susceptibility to fosmidomycin (as well as clindamycin) is an important factor in determining treatment outcome. Although most patients included in the study contracted malaria from the same area (*i.e*., Tak Province, Thai-Myanmar border), parasite sensitivity to anti-malarials can be strikingly different (Chaicharoenkul, unpublished observation). *In vitro *sensitivity data indicate that there is an intrinsically low sensitivity of *P. falciparum *isolates in Thailand to fosmidomycin [20]. Unfortunately, *in vitro *sensitivities of the parasite strains to fosmidomycin and clindamycin were not investigated in either study. This observation is supported by the pharmacokinetic data, which clearly show the complete clearance of parasitaemia with no recrudescence during the 28- day follow up period) in some individuals with comparable or even lower trough plasma concentrations of fosmidomycin/clindamycin when compared with those with recrudescent parasitaemia. Minimising fluctuations between trough and peak concentrations can be achieved by administering the drugs more frequently at six hourly intervals. Given the MIC data obtained for fosmidomycin and clindamycin, it may be possible to simulate the appropriate dose regimen for the combination despite the lack *in vitro *sensitivity data.

## Conclusion

The combination of fosmidomycin (900 mg) and clindamycin (300–600 mg) administered every six hours for a minimum of five days would constitute the lowest dose regimen with the shortest duration of treatment and which could result in a cure rate greater than 95%. Combination of fosmidomycin with another partner drug with long half-life, e.g., azithromycin, may also be an alternative regimen for treatment of multidrug resistant falciparum malaria. Further study is required to prove this supposition.

## Abbreviations

AUC: Area under the plasma concentration-time curve; Cmin-ss: Minimum plasma concentration at steady-state; Cmax: Maximum plasma concentration; Cmax-ss: Maximum plasma concentration at steady-state; Cmax-min-ss: Difference between maximum and minimum plasma concentration at steady-state; Cave-ss: Average plasma concentration at steady-state; CI: Confidence interval; CL: Total clearance; CV: Coefficient of variation; F: Bioavailability; FCT: Fever clearance time; HPLC: High performance liquid chromatography; K01: Absorption rate constant (by one-compartment model); K10: Elimination rate constant (by one-compartment model); MIC: Minimum inhibitory concentration; PCT: Parasite clearance time; tmax: Time to maximum plasma concentration; tlag: Absorption lag time; t1/2a: Absorption half-life (by one-compartment model); t1/2e: Elimination half-life (by one-compartment model); t1/2z: Terminal phase elimination half-life; Vz: Apparent volume of distribution; V: Apparent volume of distribution (by one-compartment model).

## Competing interests

The authors declare that they have no competing interests.

## Authors' contributions

IKNB was the senior investigator, pharmacokinetic data analyst and manuscript co-author. RR and SL were the site investigators and overall medical coordinators. DH reviewed the study protocol and manuscript and provided technical support. AC and VB were responsible for the analysis of pharmacokinetic samples (determination of fosmidomycin and clindamycin in plasma and urine).
